# Household health care-seeking costs: experiences from a randomized, controlled trial of community-based malaria and pneumonia treatment among under-fives in eastern Uganda

**DOI:** 10.1186/1475-2875-13-222

**Published:** 2014-06-05

**Authors:** Fred Matovu, Aisha Nanyiti, Elizeus Rutebemberwa

**Affiliations:** 1 School of Economics, Makerere University, PO Box 7062, Kampala, Uganda; 2 Policy Analysis and Development Research Institute, PO Box 16064, Kampala, Uganda; 3 Development Economics Group, Wageningen University and Research Centre, Wageningen, Holland, Netherlands; 4 School of Public Health, College of Health Sciences, Makerere University, PO Box 7062, Kampala, Uganda

**Keywords:** Household cost, Health care-seeking, Malaria, Pneumonia

## Abstract

**Background:**

Home and community-based combined treatment of malaria and pneumonia has been promoted in Uganda since mid 2011. The combined treatment is justified given the considerable overlap between the symptoms of malaria and pneumonia among infants. There is limited evidence about the extent to which community-based care reduces healthcare-seeking costs at the household level in rural and urban settings. This paper assesses the rural–urban differences in direct and indirect costs of seeking care from formal health facilities compared to community medicine distributors (CMDs).

**Methods:**

Exit interviews were conducted for 282 (159 rural and 123 urban) caregivers of children below five years who had received treatment for fever-related illnesses at selected health centres in Iganga and Mayuge districts. Data on the direct and indirect costs incurred while seeking care at the health centre visited were obtained. Using another tool, household level direct and indirect costs of seeking care from CMDs were collected from a total of 470 caregivers (304 rural and 166 urban). Costs incurred at health facilities were then compared with costs of seeking care from CMDs.

**Results:**

Household direct costs of seeking care from health facilities were significantly higher for urban-based caregivers than the rural (median cost = US$0.42 for urban and zero for rural; p < 0.0001). The same is true for seeking care from CMDs (p = 0.0038). Overall, caregivers travelled for an average of 75 min to reach health centres and spent an average of 80 min at the health centre while receiving treatment. However, households in rural areas travelled for a significantly longer time (p < 0.001 to reach health care facilities than the urban-based caregivers. Besides travelling longer distances, rural caregivers spent 150 min seeking care from health facilities compared to 30 min from CMDs.

**Conclusion:**

Time and monetary savings for seeking care from CMDs are significantly larger for rural than urban households. Thus, home and community-based treatment of child febrile illnesses is much more cost-saving for rural poor communities, who would spend more time travelling to health facilities - which time could be re-directed to productive and income-generating activities.

## Background

Community-based health care interventions under the Integrated Community-based Case Management (ICCM) model are intended to provide prompt treatment so that illness does not progress to severe levels, reduce household costs of seeking treatment, and fill the gap that may exist due to inadequacy of health facilities, particularly in remote areas [1]. Community-based interventions such as community management of malaria have been recommended and are now widespread, with the basic aim of increasing access and uptake in resource-poor settings where health worker ratios remain low and health systems weak and underfunded [2-6].

In mid 2011, Uganda adopted a policy for home and community-based treatment of malaria and pneumonia as a way of improving access to health care services for the rural poor who may lack financial resources for transportation to distant health care facilities [7]. There is however, limited evidence about the extent to which this community-based intervention reduces household health care-seeking costs, and how cost of care at community level and health facility level compare between rural and urban settings.

The objective of this paper is to investigate differences in household-level costs incurred under the home and community-based treatment of malaria and pneumonia, as opposed to obtaining care at a formal health facility in both rural and urban areas. This comparison is important to provide insights into whether community-based interventions are more relevant in rural settings with limited access to health care facilities than in urban areas.

## Methods

### Study setting

The study was conducted in Iganga and Mayuge districts in eastern Uganda, between March and June 2012. This study is part of a large, randomized, controlled trial for home and community-based combined treatment of malaria and pneumonia among under-fives within the Iganga-Mayuge demographic surveillance site (DSS) (Trial registration no: ISRCTN52966230). The entire DSS has a population of about 67,000 people, 16% of whom are children below five years [8]. The main economic activity in both districts (Iganga and Mayuge) is subsistence farming, with communities living near Lake Victoria largely involved in fishing. Households living in the urban areas are engaged in trade – mainly petty and retail trade, with a few wholesale dealers in Iganga municipality. About 90% of the DSS catchment area is rural based according to the 2002 Uganda population census classification and district maps.

Health care providers in the study areas include both public and private providers. The public health facilities include a district hospital (Iganga hospital), one health centre (HC)-IV, four HC- IIIs and 11 HC-IIs. Private providers include a number of drug shops and clinics. At the community level, there are two community medicine distributors (CMDs) per village who either deliver a combination of anti-malarials and antibiotics for uncomplicated malaria and pneumonia (intervention group) or anti-malarials alone (control group) through the home and community-based management of malaria and pneumonia programme. Under fives receive free treatment at both CMDs and public health facilities, but caregivers do incur costs for transport to the facilities.

### Description of the intervention

The randomized, controlled trial for the home and community-based treatment of malaria and pneumonia among under-fives in Iganga-Mayuge DSS comprised two arms. In the treatment group, CMDs distributed artemether-lumefantrine (AL) and amoxycillin for malaria and pneumonia, respectively. In the control group, CMDs distributed AL for malaria only. Treatment was done symptomatically. CMDs in the treatment arm were given watches and trained to count respiratory rates so as to detect possible pneumonia, based on the breathing rate of the child. If the breathing rate did not suggest presence of pneumonia, then the child would receive AL only. If a child in the control group presented with symptoms of pneumonia or other respiratory tract infections (RTI), CMDs would refer the child to a formal health facility. Severe cases of malaria in each arm would be referred to formal health facilities.

### Sampling procedure and data collection

Exit interviews were conducted at eight health facilities (four in urban and four in rural areas) drawn from both the treatment and control arm of the intervention. The caregivers interviewed were selected on the basis of a child below five years of age having been diagnosed and treated for a fever-related illness. All eligible respondents who turned up on the day of interview were enrolled. In total, 282 exit interviews were conducted at the eight health centres. Caregivers were interviewed about the direct and indirect costs incurred while seeking treatment at the facility visited. The direct costs included transport and other treatment-related costs. Indirect costs were opportunity costs of time spent while travelling to and waiting at the health centre. A semi-structured questionnaire was administered at the point of exit (health facility premises) after obtaining treatment. The interviews were conducted in the common local languages in the study areas (*Lusoga* and *Luganda)* by a team of four interviewers. Prior to the interviews, the questionnaire was piloted to test for consistency, clarity of the questions and relevance of the responses to the study objectives.

Using another tool, data on expenditure made by caregivers who visited CMDs were collected in order to compare household costs incurred by caregivers at health centres and at CMDs. A sample of 66 CMDs were randomly selected and using the register for each of the selected CMDs, five to ten children aged below five who had obtained treatment from each of the selected CMD over the past one month were randomly selected. However, in a limited number of cases, children who had visited in the past 4–8 weeks were considered because of insufficient cases recorded in the last four weeks. A total of 470 caregivers who had sought care for febrile illness from CMDs were interviewed at their homes.

### Data collection, management and analysis

Completed questionnaires were edited and coded prior to data entry. Data were entered into an Excel spreadsheet and cleaned prior to analysis. Data were analysed using STATA version 12. The analysis was based on two research questions: (1) What are the potential costs households avoid by seeking treatment for malaria and pneumonia for under-fives from CMDs as opposed to health centres? (2) How do healthcare-seeking costs compare between rural and urban settings?

The primary outcome for the study was the difference in direct and indirect costs of seeking care from health centres as compared to CMDs in rural and urban areas. Caregivers reported the amount of money they had spent on transport and other treatment-related expenditure, such as on refreshments while seeking care. The household-level direct and indirect costs incurred while seeking care from health centres were computed and compared with corresponding costs incurred by seeking care from CMDs. Drugs costs were not examined since drugs were being given free of charge whenever available, in line with current policy in Uganda to provide free medical services at public health facilities. The differences in direct and indirect costs reflect the avoided costs of care by receiving treatment from CMDs rather than from health facilities. A Wilcoxon rank sum test was performed to test for statistical significance of differences in medians of outcomes of interest between rural and urban households. Since costs were highly skewed due to a high number of respondents reporting incurring zero costs, the usual two-way sample t-test of means was not appropriate; instead an alternative test of median values was done.

### Ethical approval

Ethical approval for the main randomized trial of which this analysis is part was sought from Makerere University School of Public Health Higher Degrees Research and Ethics Committee Board and from the Uganda National Council for Science and Technology (HS 72). Permission to conduct the surveys was obtained from the Iganga and Mayuge District Health Officers. Verbal informed consent was obtained from each CMD and caretaker interviewed. Confidentiality was maintained throughout data collection, management, analysis and reporting.

## Results

### Sociodemographic characteristics of respondents

The majority of caregivers (both in the exit and household interviews) were female (96%) and married (83%). The main economic activity for households in the study areas is subsistence farming (49%) followed by petty trade (24%). Table 1 presents sociodemographic characteristics of the caregivers interviewed in the study.

**Table 1:** Sociodemographic characteristics of respondents

**Characteristics of caregiver**	**Exit interviews (n = 282)**			**Household interviews (n = 470)**		
	**Rural**	**Urban**	**Total**	**Rural**	**Urban**	**Total**
	**N (%)**	**N (%)**	**N (%)**	**N (%)**	**N (%)**	**N (%)**
**Gender** Female						
**Marital status**	153 (96.2)	118 (95.9)	271 (96.1)	277 (91.1)	157 (94.6)	434 (92.3)
Married	126 (79.3)	110 (89.4)	236 (83.7)	272 (89.5)	139 (83.7)	411 (87.5)
Widow	3 (1.4)	5 (4.1)	8 (2.8)	12 (3.9)	9 (5.4)	21 (4.5)
Never married	15 (9.4)	4 (3.3)	19 (6.7)	9 (3.0)	9 (5.4)	18 (3.8)
Divorced/separated	15 (9.4)	4 (3.3)	19 (6.7)	11 (3.6)	9 (5.4)	20 (4.3)
**Occupation of caregiver**						
Peasant farmer	110 (69.2)	29 (37.2)	139 (49.3)	260 (85.5)	15 (9.0)	275 (58.5)
Petty trader	41 (25.8)	28 (35.9)	69 (24.5)	23 (7.6)	55 (33.1)	78 (16.6)
Private/public employee	22 (13.8)	5 (6.4)	27 (9.6)	6 (2.0)	20 (12.0)	26 (5.5)
Casual worker	30 (18.8)	10 (12.8)	40 (14.2)	11 (3.6)	72 (43.4)	83 (17.7)
Unemployed	1 (0.6)	6 (7.7	7 (2.5)	4 (1.3)	4 (2.4)	8 (1.7)
**Age**						
Mean age (range) of caregiver (yrs)			26.3 (10–60)			31.7 (16–80)
Mean age (range) of child (months)			14.36 (1–60)			27.3 (1–60)

### Household-level direct costs

The average direct costs of seeking care at formal health facilities are reported in Table 2. Overall, 59% (166/282) of the caregivers who sought care from health centres reported incurring costs while seeking care. Transport costs were the most significant costs incurred, although were small in absolute terms. Average transport costs were significantly higher in the urban than in rural areas (p < 0.0001), other treatment-related costs were not significantly different (p = 0.573). Overall however, average health care-seeking costs were significantly greater for households in urban areas (p < 0.0001). Other categories of treatment-related costs comprised mostly expenditure on soft drinks, snacks or mineral water bought during the visit, and an exercise book for recording the prescription by the health worker. Drugs costs were not examined since drugs were being given free of charge whenever available. For caregivers who sought care from the CMDs, only 9% (42/470) reported having incurred any costs while seeking care. The average and median direct costs of seeking care from CMDs are reported in Table 3.

**Table 2:** Household-level direct treatment costs for seeking care at health centre

**Variable**	**Rural (n = 159)**			**Urban (n = 123)**			**Overall (n = 282)**			**p-value**
**Cost (UGX)**	**Mean**	**Median**	**SD**	**Mean**	**Median**	**SD**	**Mean**	**Median**	**SD**	
Transport	257.9	0	797.3	1,417.1	1,000	2,276.6	763.5	0	1,714.5	<0.0001
Other cost	120.1	0	350.9	108.1	0	269.7	114.9	0	317.6	0.573
Total	378.0	0	916.9	1,525.2	1,000	2,296.8	878.4		1,757.2	<0.0001
Total (US$)	0.16	0	0.38	0.64	0.42	0.96	0.37	0	0.73	<0.0001

Note (Exchange rate US$1 = UGX2,400/=)

**Table 3:** Household-level direct treatment costs for seeking care from community medicine distributors

**Variable**	**Rural (n = 304)**			**Urban (n = 166)**			**Overall (n = 470)**			**p-value**
	**Mean**	**Median**	**SD**	**Mean**	**Median**	**SD**	**Mean**	**Median**	**SD**	
Total cost (UGX)	119.4	0	593.9	392.8	62.5	1,427.0	216.0	0	980.5	0.008
Total cost (USD)	0.05	0	0.25	0.16	0.03	0.59	0.09	0	0.41	

Source: Authors’ computations using field survey data (Exchange rate: US$1 = UGX2,400/=)

Caregivers who used CMDs and reported spending any money did so to buy milk, passion fruits upon advice from CMDs to give AL to child along with such foods and refreshments while away from home. Only two caregivers reported spend on transport while receiving care at CMDs. On average, caregivers spent UGX.200 (US$0.09) while seeking care from CMDs. Households in urban areas spent about three times more than those in rural areas (p = 0.008).

### Household-level indirect costs

Table 4 shows the average indirect costs in terms of travel and waiting time spent seeking care at health centres.

**Table 4:** Opportunity costs for healthcare-seeking at the health centres by location

**Variable**	**Rural (n = 159)**		**Urban (n = 123)**		**Overall (n = 282)**	
	**Mean**	**SD**	**Mean**	**SD**	**Mean**	**SD**
Travel time (minutes)	90.4	74.0	55.7	58.9	75.3	69.9
Waiting time (minutes)	59.6	70.3	106.5	71.0	80.0	74.2
Total time (minutes)	150.0	98.9	162.1	94.0	155.3	96.8
Distance (km)	1.3	5.1	1.6	3.4	1.5	4.4

Overall, caregivers travelled for an average of 75 minutes to reach the health centres and spent up to an average of 80 minutes at the health centre while receiving treatment. Over 28% (80/282) of the caregivers travelled for 90 minutes or more to reach a health facility. Households in rural areas travelled for significantly longer (p < 0.001) but their waiting time was relatively shorter than for urban households (p < 0.001). The combined effect of travel and waiting time was not significantly different between rural and urban households (p = 0.289). In terms of distances covered, caregivers travelled an average of 1.5 km to reach a health facility. However, 12% (32/282) of the caregivers reported having travelled for more than 2 km. On average, the distances travelled by rural and urban caregivers to health facilities were not significantly different (p = 0.565).

With respect to receiving care from CMDs, caregivers travelled for an average of 20 minutes compared to 75 minutes spent travelling to receive care from health care facilities (Table 5).

**Table 5:** Opportunity costs for healthcare-seeking at community medicine distributors by location

	**Rural (n = 304)**		**Urban (n = 166)**		**Overall (n = 470)**	
**Variable**	**Mean**	**SD**	**Mean**	**SD**	**Mean**	**SD**
Travel time (minutes)	21.6	20.4	13.7	23.9	18.8	22
Waiting time (minutes)	8.9	8.2	7.4	5.9	8.4	7.5
Total time (minutes)	30.5	23.4	21.1	25	27.2	24.3
Distance travelled (km)	0.4	0.3	0.2	0.2	0.3	0.3

The waiting times were generally low with caregivers spending only eight minutes receiving care from CMDs, compared to 80 minutes at the health facilities. However, these indirect costs are likely to be subject to recall biases since some caregivers interviewed had visited the CMDs about 8 weeks prior to the date of the interview. The overall time cost (travel and waiting time) was slightly higher for rural caregivers (30 minutes) compared to urban caregivers (21 minutes) (p = 0.0001).

In terms of distance, caregivers in rural areas covered a significantly longer distance to reach CMDs (400 m) than in the urban areas (200 m) (p < 0.0001). Overall, caregivers had to cover an average distance of 300 m to reach CMDs, which was far shorter than the 1.5 km average distance to health care facilities.

## Discussion

This paper explored differences in household-level costs incurred by rural and urban households to seek treatment for febrile illness for under fives from CMDs as compared to health care facilities.

Household direct costs of seeking care from health facilities were significantly higher for urban-based caregivers than rural, and transport costs were the cost driver. Drugs at health facilities were free, at least for the under fives, however, some caregivers reported they had not received some drugs prescribed by the health worker and therefore would have to buy them from drug shops and pharmacies. About 33% of the caregivers interviewed had not received some or all drugs prescribed.

On average, caregivers from the urban areas spent significantly more on transport to reach health centres than caregivers from the rural areas. This is because people in urban areas tend to hire motor cycles (locally known as *boda bodas*) or public transport vehicles to reach health centres compared to those from rural areas who either walked or used their own (or borrowed) bicycles. On average, caregivers in the urban areas spent four times more than those in rural areas. However, the lower direct costs incurred by rural caregivers were out-matched by the significantly higher indirect costs in terms of travel time.

Whereas the monetary costs of seeking-care are small for rural households, they do incur a higher opportunity cost in travel time. Thus community-based interventions would save the rural poor valuable time, which they could then utilize in productive activity. The model for comprehensive community- and home-based healthcare (CCHBHC) was developed to ensure better accessibility to health and quality community health care, particularly for rural areas which tend to be severely underserved by formal healthcare facilities [9]. The findings of this study show that a significant amount of time is saved when caregivers obtained treatment from CMDs compared to health facilities. This is in line with the objectives of the CCHBHC model of reducing healthcare costs for the poor.

Indirect costs of facility visits were higher for rural caregivers, suggesting that seeking care from CMDs is more relevant for rural than urban areas. Moreover, access to healthcare facilities in the rural areas in the study area and other similar low-income settings tends to be poor. Thus, seeking care from CMDs by rural caregivers is cheaper and likely to ensure more prompt management of illness and limit their progression to severe levels.

Since rural households tend to be poorer [10], seeking care for febrile illness also saves them incurring out -of-pocket expenses on a limited income. This finding is consistent with that of [11] who reveal that children from households with higher socio-economic status were more likely to seek healthcare at health facilities. This implies that the rural poor households stand a higher risk of not seeking care from health facilities due to the monetary cost involved. Evidence from other studies also shows that cost is a significant barrier to health access, especially for rural communities [12,13]. The results in Table 2 and 3 show that the direct and indirect cost of obtaining treatment from health facilities were over four times higher than from CMDs. The results of this study further show that the use of CMDs, especially for rural caregivers, significantly reduces the household costs of seeking care. To the extent that introduction of combined treatment of malaria and pneumonia at CMDs leads to more rural households visiting CMDs instead of facilities, then the average cost per episode would fall.

The analysis of the socio-demographic characteristics shows that children who were taken to health facilities were relatively younger compared to those taken to CMDs. This could imply that mothers/caretakers prefer to have a more comprehensive diagnosis for infant illness with a more qualified health worker than taking them to the less qualified CMDs. This implies that uptake of community-based health interventions is likely to be higher for older infants.

## Conclusion

The direct costs of seeking care from health facilities were significantly higher for urban-based caregivers than for those in rural areas. Similarly the direct costs of seeking treatment from health facilities was four time higher than from CMDs. Whereas the monetary costs of seeking care are small for rural households, they do incur a higher opportunity cost in travel time. Thus community-based interventions would be likely to save the rural poor valuable time, which they could then utilize in productive activity. Seeking care from CMDs by rural caregivers is both cheaper and likely to ensure more prompt management of illness and limit their progression to severe levels. Therefore, home and community-based interventions should be promoted, particularly targeting rural areas, which tend to be underserved by formal health facilities, resulting in significantly high costs of care for the rural low-income populations.

## Abbreviations

AL: Artemether lumefantrine; CCHBHC: Comprehensive Community- and Home-based Health Care; CMD: Community Medicine Distributors; ICCM: Integrated Community Case Management; HC: Health Centre.

## Competing interests

The authors declare that they have no competing interests.

## Authors’ contributions

FM led the drafting of this manuscript, supervised data collection and analysed the data. AN helped with data collection and analysis and co-wrote the paper. ER conceptualized the main trial, helped with interpretation of results and revision of the manuscript. All authors read and approved the final manuscript.
